# Co-research in the development of AI and digital health tools for cancer management and care: a systematic review

**DOI:** 10.1186/s12913-026-14620-0

**Published:** 2026-04-30

**Authors:** Carina Dantas, Carla Reigada, Miriam Cabrita, Alexandros Mitsis, Cristina Ghiță, Marius Geanta, Félix Villanueva, Ikechukwu Nkisi-Orji, Tuukka Hakkarainen, Georgia Karanasiou, Dimitris Fotiadis, Ana Sofia Carvalho, Elísio Costa

**Affiliations:** 1 SHINE 2Europe Lda, Coimbra, Portugal; 2 ICBAS – School of Medicine and Biomedical Sciences, University of Porto, Porto, Portugal; 3 UNIR - Universidad Internacional de la Rioja, Logroño, Spain; 4 Department of Materials Science and Engineering, University of Ioannina, Ioannina, Greece; 5 INOMED - Centre for Innovation in Medicine, Bucharest, Romania; 6 Computer Architecture and Networks Group, University of Castilla–La Mancha, Ciudad Real, Spain; 7 School of Computing, Robert Gordon University, Aberdeen, Scotland; 8 Nordic Healthcare Group, Espoo, Finland; 9 RISE-Health, Faculty of Pharmacy and Competences Centre on Active and Healthy Ageing (Porto4Ageing), University of Porto, Porto, Portugal

**Keywords:** Patient and public involvement, Co-research, Artificial intelligence, Cancer, Oncology, Participatory action research

## Abstract

**Background:**

Participatory approaches (including co-research, co-design, Patient and Public Involvement [PPI], and Participatory Action Research [PAR]) are increasingly being incorporated into digital health innovation. However, their application in cancer-related digital and Artificial Intelligence (AI) tool lifecycle remains insufficiently synthesised, limiting understanding of how participatory approaches influence ethical outcomes, trust, and the real-world implementation of these technologies. This review moves beyond documenting participatory approaches to critically examine how far participation extends across the AI lifecycle, and how this limits ethical and trust-related outcomes.

**Objective:**

To examine how participatory approaches are used in the lifecycle of digital and AI-based tools for cancer care, and to analyse their ethical and trust-related impacts.

**Methods:**

A systematic review was conducted following JBI methodology. Searches of PubMed, Embase, Scopus, and ScienceDirect identified studies published in English between 2010 and 2025. The inclusion criteria required empirical research reporting the participatory involvement of patients, survivors, caregivers, or public contributors in the design, development, implementation, or governance of digital or AI tool development for cancer care. Quality appraisal was performed using the CASP checklist. Data were synthesised using a meta-aggregation approach and organised into thematic domains.

**Results:**

Of 2,742 records identified, 40 studies met the inclusion criteria. The largest proportion was published in 2024. Co-design emerged as the most frequently used participatory approach, particularly in early development stages (e.g., requirements identification, content co-creation, usability testing), while participatory involvement in later stages, such as implementation, auditing, or governance, was limited. Participation centred primarily on patients/survivors and clinicians, with caregivers and policy-level stakeholders comparatively under-represented. Most studies focused on general digital health technologies such as mobile apps and telehealth, with relatively few addressing AI-specific development components such as model training, validation, or explainability. Very few studies reported explicit measurements of ethical outcomes such as trust, fairness, or transparency.

**Conclusions:**

Participatory approaches are widely referenced but remain predominantly concentrated in early design phases of digital tool development. Future research should extend co-research into later lifecycle stages, include broader stakeholder groups beyond patients and clinicians, and systematically capture ethical and trust-related outcomes. These shifts are necessary to move beyond symbolic participation toward demonstrable and equitable impact in the development of digital and AI-enabled technologies for cancer care.

**Supplementary Information:**

The online version contains supplementary material available at 10.1186/s12913-026-14620-0.

## Introduction

Artificial Intelligence (AI) is increasingly recognised for its potential to enhance cancer diagnosis, prognosis, and treatment planning, and forms part of a broader ecosystem of digital health technologies, including mobile health applications, clinical decision-support systems, patient-reported outcome platforms, and telehealth solutions [1–3]. These innovations are reshaping cancer care by improving efficiency, personalisation, and access to services. However, their rapid development has also raised important concerns related to ethical and trust-related outcomes, including algorithmic bias, lack of transparency, limited accountability, and the exclusion of patient and public perspectives in design and implementation processes [4–7].

Participatory approaches have been proposed to address these concerns by incorporating experiential knowledge and promoting more inclusive, trustworthy, and accountable innovation. These approaches include Patient and Public Involvement (PPI), Participatory Action Research (PAR), co-research, and co-design, which differ in scope but share a commitment to involving patients, caregivers, and other stakeholders in shaping research and innovation processes. In this study, we use the term “participatory approaches” as an overarching framework to encompass these related methodologies [8–12].

Existing literature suggests that participatory approaches can improve the relevance, acceptability, and ethical and trust-related outcomes of digital health interventions, particularly by aligning technological development with user needs and values [8–15]. However, their application in AI-enabled cancer care remains insufficiently understood. Current research has largely focused on early stages of development, particularly design and usability, often through co-design practices centred on user experience [8–15]. In contrast, later stages of the technology lifecycle such as implementation, evaluation, and governance, have received comparatively limited attention. This aligns with the INVOLVE definition of Patient and Public Involvement (PPI), which emphasises research conducted ‘with’ or ‘by’ members of the public rather than ‘to’ or ‘for’ them. Such involvement is particularly relevant in research and innovation processes, as it enables earlier identification of concerns and more timely resolution of acceptability challenges [10–13].

Importantly, participatory involvement in more technically complex stages of AI development may be more difficult to implement in practice [4–7, 16, 17]. Processes such as data governance, model development, validation, and ongoing oversight require specialised technical expertise and involve methodological and regulatory constraints that may limit the extent and form of stakeholder participation [16, 17]. These challenges highlight the need to critically examine not only where participatory approaches are applied, but also where they remain limited across the AI lifecycle. At the same time, there is growing interest in ethical and trust-related outcomes in AI in healthcare, particularly in relation to fairness, transparency, accountability, and trust [4–7]. While these dimensions are widely discussed at a conceptual level, there is limited empirical evidence on how they are operationalised in practice, especially in the context of participatory research and innovation [4–15]. Understanding how participatory approaches contribute to or fail to address, these outcomes are therefore essential.

This review addresses these gaps by examining how participatory approaches are applied across different stages of the lifecycle of digital and AI-based tools for cancer care, and by analysing their implications for ethical and trust-related outcomes. By moving beyond a narrow focus on design and usability, this study aims to provide a more comprehensive understanding of participation across the full innovation process.

To support this analysis, we distinguish between general digital health stages (e.g., needs assessment, design, usability testing) and AI-specific stages (e.g., data curation, model development, validation, implementation, and governance), as these involve different technical, ethical, and organisational considerations and shape opportunities for participatory involvement [16, 17].

## Methods

This systematic review was conducted in accordance with the Joanna Briggs Institute methodology for systematic reviews of qualitative evidence [18]. The study protocol was registered under PROSPERO 2025 CRD420251171270.

The objective of the review was to synthesise the available literature on participatory approaches applied across the lifecycle of digital and Artificial Intelligence (AI)–based tools for cancer care, with a particular focus on their implications for ethical and trust-related outcomes.

### Literature search strategy

The search strategy was developed by the research team based on the review objectives and informed by existing literature and methodological guidance. The search strategy was piloted and refined prior to final implementation and was conducted across the electronic databases PubMed, Embase, Scopus, and ScienceDirect. As detailed in Table 1, the search strategy combined Medical Subject Headings (MeSH) and free-text terms encompassing three conceptual domains: participatory methodologies (e.g., “patient and public involvement,” “PPI,” “co-research,” “participatory research,” “co-design,” and “stakeholder engagement”); digital and artificial intelligence–based technologies (e.g., “artificial intelligence,” “machine learning,” “digital tool,” and “digital health”); and cancer-related contexts (e.g., “cancer” and “oncology”). Digital health and AI were intentionally combined to capture the broader technological ecosystem in cancer care. However, distinctions between general digital health tools and AI-specific applications were maintained throughout the review process to ensure conceptual clarity.

**Table 1:** Search strategy for PubMed

Terms and queries
“patient and public involvement“[Title/Abstract] OR PPI[Title/Abstract] OR "participatory research“[Title/Abstract] OR "participatory action research“[Title/Abstract] OR "co-research“[Title/Abstract] OR "co-production“[Title/Abstract] OR “co-design“[Title/Abstract] OR "user involvement“[Title/Abstract] OR “stakeholder engagement“[Title/Abstract])
“artificial intelligence“[MeSH Terms] OR “machine learning“[MeSH Terms] OR "deep learning“[Title/Abstract] OR "algorithm*“[Title/Abstract] OR AI[Title/Abstract] OR "intelligent system*“[Title/Abstract] OR "digital tool*“[Title/Abstract] OR "digital health“[Title/Abstract] OR "mobile application*“[Title/Abstract] OR "mobile health“[Title/Abstract] OR mHealth[Title/Abstract] OR eHealth [Title/Abstract] OR "telehealth“[Title/Abstract] OR "digital intervention*“[Title/Abstract] OR "decision support system*“[Title/Abstract]
(“cancer“[MeSH Terms] OR "oncolog*“[Title/Abstract] OR "neoplasm*“[Title/Abstract] OR "cancer care“[Title/Abstract] OR “oncologic care“[Title/Abstract]

### Eligibility criteria

Studies were identified through structured database searches and screened using predefined inclusion and exclusion criteria in a systematic and transparent manner.” Studies were included if they involved cancer patients, survivors, caregivers, or public contributors as active collaborators or stakeholders in the research process. Eligible studies employed participatory approaches (including PPI, PAR, co-research, co-design, or related methodologies) in the design, development, implementation, or evaluation of digital or AI-based tools for cancer care. Also, studies were included across different stages of the technology lifecycle, including design, development, implementation, and governance. Only empirical studies (qualitative, quantitative, or mixed-methods) published in peer-reviewed journals in English between January 2010 and October 2025 were considered.

Studies were excluded if they were non-empirical in nature (e.g., editorials, commentaries, or opinion pieces), if they did not include participatory involvement, did not focus on digital or AI technologies in oncology, and were not written in English.

### Study selection process

All retrieved records were imported into Rayyan, and duplicates were removed. Two independent reviewers screened titles and abstracts against the inclusion criteria. Full texts of potentially eligible studies were assessed independently by the same reviewers. Disagreements were resolved through discussion or consultation with a third reviewer. The study selection process was documented using a Preferred Reporting Items for Systematic Reviews and Meta-Analyses (PRISMA) flow diagram.

### Data extraction

Data extraction was conducted using a structured extraction form based on the Joanna Briggs Institute (JBI) methodology, designed to capture comprehensive information from each included study [18]. Extracted data included study identification details such as the title, authors, year of publication, and country of origin. Additionally, information regarding the study design, research objectives, and methodological approach was captured. Participant characteristics, including demographics and relevant contextual factors were noted to allow for an in-depth understanding of the study population.

The technological context of each study was also captured and classified as either general digital health tools or AI-specific applications. This distinction enabled comparative analysis across different types of technologies within the broader ecosystem. In addition, data extraction captured how participatory approaches were applied across different stages of the technology lifecycle and how these were reported to influence ethical and trust-related outcomes. Participatory approaches were identified based on both explicit terminology (e.g., PPI, PAR, co-design) and reported descriptions of stakeholder involvement. Studies were considered to include participatory approaches where they demonstrated active involvement of patients, caregivers, or other stakeholders in shaping the research or development process (e.g., through contributions to design, decision-making, or evaluation), even where such approaches were not explicitly labelled.”

### Quality appraisal

The methodological quality of included studies was assessed using the Critical Appraisal Skills Programme (CASP) tool [15]. Appraisal was conducted independently by two reviewers, with discrepancies resolved through discussion. Each study was evaluated against the relevant checklist. In line with CASP guidance, the tool was applied qualitatively to assess methodological rigour across studies. The appraisal was used to identify strengths and limitations within the evidence base and to inform the interpretation of findings during synthesis. Quality appraisal informed the interpretation of findings but was not used as a basis for exclusion.

### Data synthesis

Data synthesis was conducted in accordance with PRISMA guidance for systematic reviews and qualitative evidence synthesis, using a meta-aggregation approach to integrate findings across the included studies. Extracted qualitative data (e.g., authors’ interpretations, participant-reported outcomes, and descriptions of participatory processes) were systematically coded and grouped into categories based on similarity of meaning, from which overarching analytical themes were inductively derived [20].

The synthesis was guided by review questions focusing on (1) how participatory approaches are applied across different stages of the lifecycle of digital and AI-based tools for cancer care, and (2) how these approaches are associated with ethical and trust-related outcomes. Ethical dimensions were analysed as outcomes emerging from participatory processes rather than as independent analytical constructs.

Particular attention was given to how stakeholder involvement was described as influencing trust, transparency, inclusivity, and accountability. Where relevant, findings were examined separately for general digital health tools and AI-specific applications to preserve conceptual distinctions while enabling integrated interpretation.

In line with qualitative synthesis reporting standards, the analysis also examined how co-research experiences were reported, enabling participants to critically assess, monitor, and shape digital technologies in oncology settings. This structured yet inductive approach supported the identification of convergent findings across diverse contexts while preserving context-specific details, thereby enhancing the credibility, transferability, and interpretive depth of the synthesis.

During analysis, an initial framework was developed through line-by-line coding of the extracted qualitative findings from the included studies. Coding and theme development were conducted iteratively, with regular discussions between two primary reviewers and a third senior reviewer. Through this process, three overarching analytical domains were identified: (1) forms and stages of participation, (2) participant roles and experiences, and (3) implications for ethical and trust-related outcomes. Preliminary categories were reviewed and refined across multiple rounds of discussion until consensus was reached.”

## Results

**Fig. 1:**
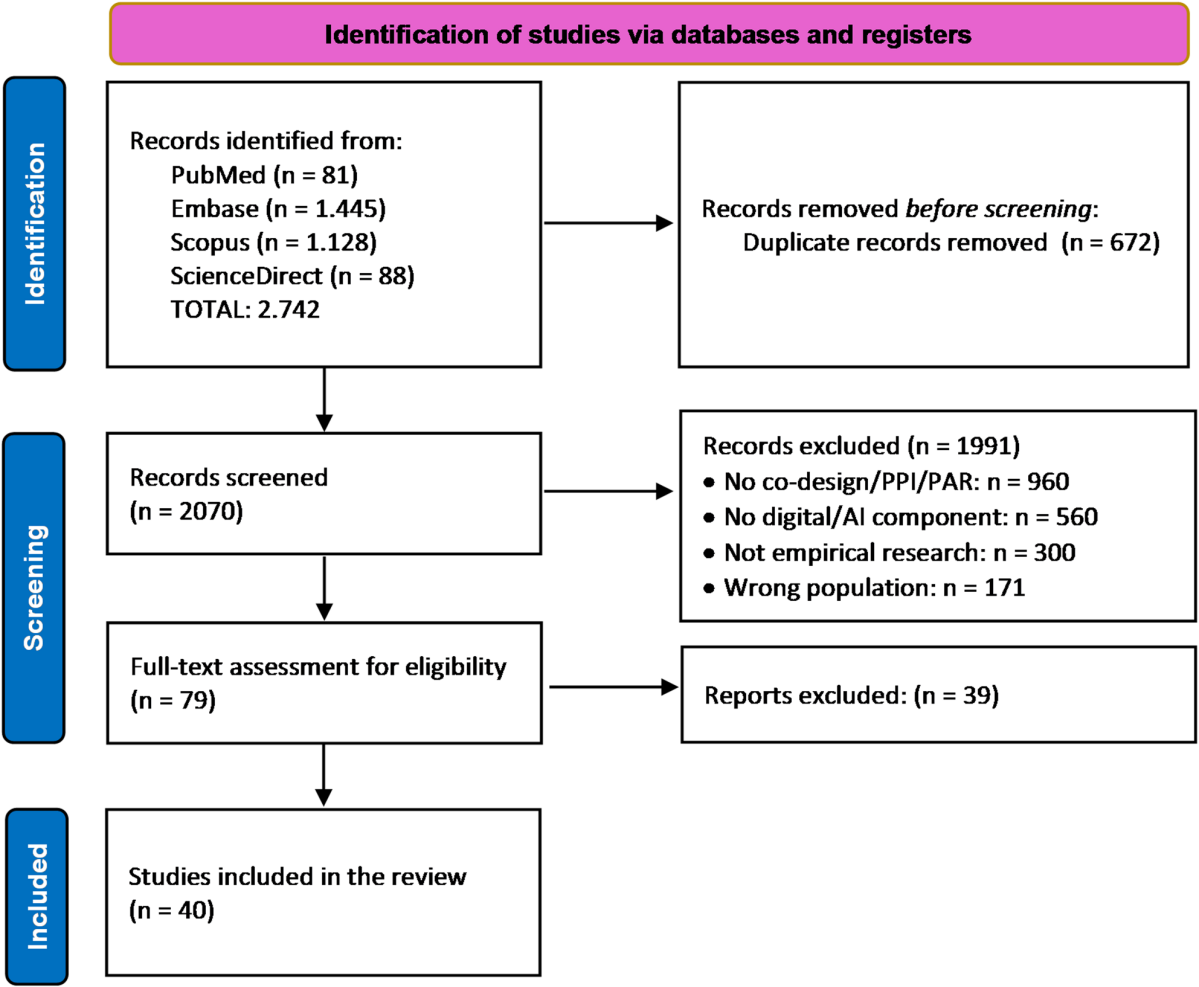
PRISMA flow diagram

A total of 2,742 records were found through database searching. Following the removal of duplicates, 1,991 records were excluded during the title and abstract screening process. Seventy-nine articles were kept for full-text assessment. Full-text screening was conducted independently by two reviewers per article, with disagreements resolved through discussion or consultation with a third reviewer. This process resulted in the inclusion of 40 studies that met the eligibility criteria. The study selection process is presented in a PRISMA flow diagram (as shown in Fig. 1).

### Use of AI and digital technologies

Although the search strategy included terms related to Artificial Intelligence (AI), the included studies encompassed both AI-specific applications and general digital health tools. Only a small number of studies (*n* = 2, 5%) explicitly engaged with AI-related or data-driven methodologies, including data-driven decision support and natural language processing applied to real-world cancer data [22, 31]. A further subset of studies (*n* = 3, 7.5%) described digital systems that could support algorithmic or automated decision-making but did not explicitly report the use of AI methods [22, 27, 31].

The majority of included studies (*n* = 35, 87.5%) focused on general (non-AI) digital health technologies, such as mobile applications, telehealth platforms, web-based interventions, and chatbot-based systems [19, 21, 24, 27–30, 33–38, 40–42, 44]. These findings indicate that participatory research in cancer-related digital health remains predominantly centred on conventional digital tools rather than advanced AI applications.

The final set of 40 studies reflects a diverse range of participatory digital health initiatives in oncology, including exercise toolkits for cancer survivors [30], chatbot-based informational and emotional support systems [19], mobile health solutions for caregivers of paediatric cancer patients [21, 34, 37, 44, 45], co-created palliative care applications [22, 27], symptom-reporting tools and patient-reported outcome dashboards [33, 35, 36], and culturally tailored digital interventions [25, 28, 46].

The entire selection process, from initial identification through screening, eligibility assessment, and final inclusion, is presented in a PRISMA flow diagram to ensure methodological transparency and reproducibility.

The thematic analysis was organised around three primary domains as shown in Table 2: (a) participants’ experiences and perspectives, (b) ethical considerations associated with participatory approaches, and (c) reported impacts on the development and implementation of digital and AI-based tools. The results below are structured accordingly. Most of the included studies documented some form of participatory or stakeholder engagement in shaping digital tools, particularly during early design phases.

**Table 2:** Distribution of included studies across stages of the digital and AI technology lifecycle

Lifecycle stage	Number of studies (*n*, %)	Example references
Design and early development	32 (80%)	[21–25, 27–36, 40–42]
Implementation and deployment	10 (25%)*	[27, 28, 35, 38, 39]
Governance and post-deployment monitoring	0 (0%)*	—

*Categories are not mutually exclusive; some studies addressed multiple stages of the lifecycle

### Participants’ experiences and perspectives

#### Co-research and participatory approaches

Co-design or co-creation emerged as the predominant participatory approach, reported in 23 of 40 studies (57.5%). These studies spanned a range of contexts, including survivorship, self-management, symptom monitoring, caregiver support, and decision support [21–34, 36, 38, 40–42, 47, 48, 49]. This prevalence supports the characterisation of co-design as the dominant participatory approach in cancer-related digital health research.

A further subset of studies (*n* = 10, 25%) also adopted broader stakeholder-driven or participatory development approaches without consistently using the co-design label, including telehealth programmes and patient-reported outcome systems [35, 36, 38]. Approaches aligned more closely with participatory action research or community-based participatory research were less common (*n* = 4, 10%) and were primarily observed in studies targeting underserved or culturally specific populations [25, 28].In contrast, approaches aligned more closely with classical participatory action research or community-based participatory research were uncommon. These approaches were primarily featured in studies targeting underserved or culturally specific populations, such as the Hmong eHealth website or culturally tailored mobile applications [25, 25]. This pattern suggests that, while participation is present across the field, it is most frequently operationalised through design-oriented methods rather than longer-cycle action research.

#### Stakeholders involved

Patients and cancer survivors were involved in the studies, often alongside clinicians and healthcare professionals. Many co-design studies focused on women after breast cancer treatment, breast cancer survivors, or adults living with and beyond cancer [24, 28, 30, 32, 38, 42]. Other studies involved parents and caregivers of children with cancer [21, 34, 37, 44].

Clinicians were frequently involved (*n* = 25, 62.5%), particularly in co-designing decision-support or symptom-monitoring tools [25, 33]. Caregivers appeared less consistently (*n* = 10, 25%) and were mainly included in studies focused on caregiver-specific tools [21, 34, 37]. Policy-level stakeholders were involved in fewer than five studies (*n* = 4, 10%). Policy-level actors or system-level decision-makers were rarely involved and primarily appeared in more programmatic, multi-stakeholder initiatives, such as telehealth services or large-scale patient-reported outcome implementations [35, 38].

### Ethical considerations and implications

#### Stage of development

Most interventions (*n* = 32, 80%) were situated at early stages of the technology lifecycle (e.g., design, development, usability testing, or pilot phases) [21–25, 27–33, 34–36, 40–42]. A subset of studies (*n* = 10, 25%) also addressed implementation or related challenges, including telemedicine during the COVID-19 pandemic [28], platform implementation [27], and large-scale initiatives such as ePRO systems [35, 38].This distribution suggests that participatory work in this domain is primarily concentrated in early development phases rather than later-stage implementation or routine clinical use. Notably, no studies reported specifically on participatory involvement in governance or post-deployment monitoring stages.

#### Cancer types and populations

Breast cancer was the most frequently represented condition (*n* = 12, 30%) [24, 28, 30, 32, 33, 36, 38]. Other cancer types included brain tumours [25], oesophageal cancer [36], ovarian cancer [39], prostate cancer survivorship [40], childhood cancer involving parents and caregivers [20, 34, 37], and multiple myeloma [29]. Several studies (*n* = 12, 30%) focused on mixed or unspecified cancer populations [15, 23, 27, 31, 35, 40–42].

### Impact on digital and AI tool development

#### Technologies

Most included studies (*n* = 35, 87.5%) focused on general digital health technologies such as mobile applications, web-based platforms, telehealth services, chatbots, or text-message interventions, as shown in Table 3. Only one study (*n* = 1, 2.5%) explicitly referred to AI-related approaches in its aims [22]. None of the 40 studies reported the development or evaluation of large language models or generative AI systems. Taken together, these findings suggest that AI, in its strict technical sense, remains relatively uncommon within participatory cancer digital health research, and that co-design is currently more frequently applied to non-AI digital interventions than to AI-specific development.

**Table 3:** Synthesised summary of participatory trends, ethical dimensions, and technological implications in cancer-related digital health research

Domain	Theme	Key Findings
Participants’ experiences and perspectives	Co-research & participatory approaches	55% of studies used explicit co-design/co-creation terminology; participation mostly operationalised through design collaboration rather than long-cycle participatory action research; culturally tailored participatory approaches used mainly for underserved groups.
	Stakeholders involved	Most involved participants were patients/survivors and clinicians; caregivers were less frequently included and primarily in paediatric/caring contexts; policy-level/system-level stakeholders rarely participated.
Ethical considerations and implications	Stage of development	Majority of studies occurred in early phases: design, development, usability testing; limited evidence of later-stage implementation or sustained use in real-world clinical settings.
	Cancer types and populations	Breast cancer most frequently represented; additional groups included paediatric oncology, brain tumours, oesophageal, ovarian, prostate cancer and multiple myeloma; several studies used non-tumour-specific populations (e.g., general survivors).
Impact on digital and AI-tool development	Technologies	Most tools were conventional digital health solutions (apps, web systems, telehealth, messaging); only one explicitly referenced data-driven decision-support ethics; no studies involved LLMs or generative AI systems.

## Discussion

This review aims to describe the use of co-research approaches in the development of digital and AI-related tools for cancer care, and to assess the extent to which these approaches facilitate meaningful, multi-stakeholder participation. Findings from the 40 included studies indicate a landscape in which participatory methods are widely invoked but unevenly integrated. Participation is predominantly concentrated in early design stages, with far more limited engagement during later phases of implementation, data governance, or algorithmic development.

### Co-design as the default mode

The predominance of co-design across diverse contexts, including survivorship, caregiver support, culturally tailored interventions, symptom monitoring, and decision-support tools, indicates that co-design has become the primary participatory methodology in cancer-related digital health research [21–34, 36, 38, 40–42, 48]. While this development is encouraging and reflects growing recognition of participatory principles, co-design is often used primarily to structure discussions around user needs, acceptability, and usability, rather than to redistribute decision-making authority over technical, organisational, or governance aspects of digital systems [44]. Participation is therefore strongest in front-end phases of development, with limited involvement in later stages where issues such as bias mitigation, data governance, auditability, and accountability become critical.

### Limited reach into AI-specific processes

The limited reach of co-research into AI-specific processes raises important questions regarding the drivers of this gap. Although limited technical literacy among patients and public contributors is frequently cited as a barrier to participation in AI development, our findings suggest that this explanation is insufficient on its own [4]. The absence of participatory engagement at organisational and societal levels also reflects a lack of participatory literacy within AI development cultures, where governance, data stewardship, and accountability mechanisms remain largely closed to non-technical stakeholders [4–7, 50–59]. Consequently, key decisions regarding training data selection, model validation, and performance criteria are often made without the input of those most affected by AI-mediated outcomes. This has direct implications for algorithmic bias, as unexamined data practices may reproduce existing inequities, obscure representational gaps, or privilege dominant clinical perspectives [43]. In this context, claims of “trustworthy AI” are difficult to sustain, as trustworthiness cannot be achieved through technical optimisation alone but requires inclusive and transparent participatory processes embedded throughout the AI lifecycle.

This pattern can be partly explained by a combination of structural and organisational constraints. The technical complexity of AI systems often limits participation beyond design and usability phases, while regulatory and governance requirements related to data protection, accountability, and intellectual property restrict stakeholder involvement in validation, auditing, and oversight processes [4–7, 43, 50–59]. Power asymmetries between developers, researchers, and end users further shape participatory boundaries, and organisational constraints frequently impede sustained engagement across later stages of the AI lifecycle [6, 43].

### Ethical and trust-related implications of participatory methods

Although most studies framed participation to improve acceptability, relevance, or human-centredness [1, 6–9, 12–20, 23, 25, 36–38, 42, 43], few explicitly evaluated ethical outcomes such as fairness, transparency, accountability, or trust. Instead, evaluation was predominantly limited to user experience–related constructs, including usability, acceptability, feasibility, and perceived usefulness [11, 21–23, 30, 33, 36]. While these measures provide valuable insights into interaction and uptake, they do not capture whether participatory engagement influences ethically salient dimensions of digital and AI systems. In particular, the lack of stakeholder involvement in decisions regarding training data selection and model validation constrains the assessment of algorithmic bias and equity risks [6, 43]. In the absence of systematic evaluation, participatory involvement risks becoming procedural rather than substantive, with limited evidence that it shapes safety, governance, or accountability decisions [43, 48]. As highlighted in AI ethics literature, trustworthiness cannot be inferred from positive user experience alone but requires explicit attention to fairness, transparency, and accountability across the AI lifecycle [4–7, 50–59]. Caregivers are also under-represented across the reviewed literature, appearing mainly in studies explicitly focused on caregiver-facing tools, despite their central role in cancer care, indicating a further gap for future participatory research agendas [6, 36, 38].

### Early-phase clustering

A consistent pattern across the included studies was the strong concentration on design, prototyping, and preliminary usability testing, with comparatively few examples of sustained implementation or service-level transformation [28, 29, 32, 35, 40]. Ethical challenges associated with AI and digital health frequently require organisational readiness, regulatory alignment, and long-term monitoring, which may partly explain the limited participatory engagement at these later stages [43]. In practice, researchers appear to prioritise phases that are more tractable, co-creating tools with users, while more complex institutional and systemic dimensions, such as AI oversight, auditing, interoperability, consent for secondary data use, and regulatory compliance, progress more slowly [4–7, 55–59].

### Geographic and contextual perspectives

Most tools were developed in high-income, predominantly Anglophone contexts (Australia, the United Kingdom, and North America), even when designed for culturally specific communities, such as the Hmong eHealth platform [27]. Regulatory frameworks, data-sharing norms, and institutional infrastructures differ substantially across regions, meaning that solutions developed within one jurisdiction may not be readily transferable to another [43]. This highlights the need for cross-regional validation of participatory models to ensure that digital and AI-enabled innovations do not inadvertently reinforce, rather than mitigate, global health inequities.

An additional concern emerging from this review is the risk of symbolic participation, or tokenism, whereby co-research serves primarily to legitimise projects without meaningfully influencing technical, ethical, or organisational decision-making. In several studies, participatory activities were reported without clear evidence that stakeholder input shaped data practices, algorithmic choices, or governance arrangements, suggesting a largely procedural form of participation that leaves existing power structures intact [6, 43, 48]. Only transformative participation, in contrast, has the potential to redistribute decision-making authority and contribute to more equitable and trustworthy AI systems across the technology lifecycle [4–7, 59].

### Model for future practice

The Horizon Europe–funded MAYA project (Smart Mirrors supporting healthier lives of Adolescents and Young Adults after cancer) is presented here as an explanatory case and proof-of-concept for how co-research and participatory governance can be operationalised across the lifecycle of digital and AI-enabled health technologies. More specifically, MAYA provides a concrete illustration of how the gaps identified in this review, particularly the limited reach of co-research into AI-specific processes discussed in Section “Limited reach into AI-specific processes”, can be addressed in practice. The project brings together 16 partners across 11 countries to support young cancer survivors in managing long-term cardiovascular health following cardiotoxic cancer treatments through digital tools. Central to MAYA is a participatory ethos grounded in the recognition that sustainable and ethically robust innovation emerges not from consultation alone, but from the active positioning of affected individuals as co-architects of research and development processes.

MAYA adopts a co-research framework in which five people with lived experience on cancer are formally integrated as researcher-partners across the full research lifecycle. In contrast to the early-stage, design-focused participation commonly identified in the reviewed literature (see Section  “Co-design as the default mode” and “Limited reach into AI-specific processes”), this approach extends involvement to agenda-setting, interpretation of findings, and deliberation on implementation priorities. By embedding lived experience within decision-making structures, MAYA operationalises ethical outcomes such as transparency, accountability, and trust, that were rarely reported explicitly in the studies included in this review (see Section “Early-phase clustering”).

During its initial six months, the consortium issued an open call for co-researchers, conducted a transparent selection process, and embedded these partners within all project phases. This depth of engagement directly responds to the risks identified in Section “Limited reach into AI-specific processes”, particularly those relating to algorithmic bias and opaque data practices, by creating structured opportunities for co-researchers to engage with questions of data use, digital monitoring, and decision-making logic. Ethical and trust-related dimensions will be systematically examined and documented through workshops and reflective activities with cancer survivors, patients, and other stakeholders from December 2025 onwards.

Although patients were not involved in the development of the present review protocol, the findings of this review have directly informed the participatory design of MAYA. In this way, the project exemplifies how evidence synthesis can be translated into practice by deliberately embedding mechanisms to capture and report ethical and trust-related outcomes, addressing limitations identified across the literature (see Section “Limited reach into AI-specific processes” and “Geographic and contextual perspectives”). As such, MAYA offers a transferable model for moving beyond symbolic participation toward participatory governance in the development of digital and AI-enabled tools for cancer management and care.

These findings have clear practical implications for researchers, developers, healthcare professionals, policymakers, and funders, underscoring the need to embed co-research across the full lifecycle of digital and AI-enabled technologies rather than limiting participation to early design stages. Extending participatory practices into development, validation, implementation, and governance phases is critical to strengthening ethical robustness, mitigating algorithmic bias, and supporting more trustworthy AI in oncology [4–7, 41, 57, 58]. While the MAYA project is context-specific, the participatory design choices it adopts, including early integration of lived experience partners, structured involvement in data and governance discussions, and systematic documentation of ethical and trust-related outcomes, illustrate transferable principles that can inform participatory AI governance in other oncology and digital health contexts.

### Limitations

The findings of this study should be interpreted considering several limitations. The identification and classification of participatory approaches relied exclusively on authors’ descriptions of their methods. Terminology such as “co-design,” “participatory research,” “co-production,” and “patient and public involvement” was not used consistently across studies. In the absence of detailed methodological reporting, there is a risk of both overestimating and underestimating the depth of participation. Furthermore, given the rapid evolution of artificial intelligence technologies and participatory standards in health research, this analysis should be viewed as a baseline characterisation of current practices in participatory AI within oncology, rather than a definitive account of future developments. This review also has limitations related to the review process. Although a comprehensive search was conducted across multiple databases, the choice of search terms and restriction to English-language publications may have resulted in the omission of relevant studies. Additionally, variability in how participatory approaches and AI are defined and reported in the literature may have influenced study identification.

## Conclusions

This review highlights three key priorities for advancing participatory approaches in the development of digital and AI-supported tools for cancer care. First, co-design needs to be carried forward into the later phases of AI and digital-tool development, such as ongoing monitoring, audit and update cycles, rather than only used at the early development phase. Second, stakeholder sets must be widened beyond patients and clinicians to include caregivers, developers, and policy actors and other system-level contributors, as sources of bias and exclusion frequently emerge at organisational and socio-technical levels of the value chain. Third, future participatory studies should start to report ethical and trust-related outcomes routinely, enabling co-research in cancer care to progress from aspirational engagement to demonstrable impact, aligned with ethics-by-design frameworks and emerging international guidance on AI in health. The case study of the MAYA project is a practical example of how these key observations are already being considered and implemented and may be used as inspiration for future works.

## Electronic Supplementary Material

Below is the link to the electronic supplementary material.

Supplementary Material 1

## Data Availability

The dataset(s) supporting the conclusions of this article is(are) included within the article (and its additional file(s)).

## Abbreviations

AI: Artificial Intelligence
CASP: Critical Appraisal Skills Programme tool
INVOLVE: Program of the National Institute for Health Research, to support active public involvement
JBI: Joanna Briggs Institute
PPI: Patient and Public Involvement
PAR: Participatory Action Research
PRISMA: Preferred Reporting Items for Systematic Reviews and Meta-Analyses
R&I: Research and Innovation
MAYA: Smart Mirrors supporting healthier lives of Adolescents and Young Adults after cancer Project
