# Supplementary material for: Co-research in the development of AI and digital health tools for cancer management and care: a systematic review
Source: BMC Health Serv Res. 2026 Apr 30;26:844. doi: 10.1186/s12913-026-14620-0 (PMC13277194; doi:10.1186/s12913-026-14620-0)
Supplement: Supplementary file 1 — Supplementary Material 1 [file 12913_2026_14620_MOESM1_ESM.docx]

| **Article n** | **First Author /Year** | **Study type** | **Country** | **Study objective** | **Cancer type** | **AI category** | **Co-research type** | **Involvement level** |
| --- | --- | --- | --- | --- | --- | --- | --- | --- |
| 1 | Kabukye, J. K. et al. (2023) | Qualitative study with supplementary descriptive survey (SUS) and field observations. | Sweden | Describe stakeholders’ perceptions (nurses, administrators, patients) of a smartphone-based telemedicine system (with an AI image-analysis model) to improve cervical cancer screening in Uganda; explore usability, barriers, and facilitators of adoption. | Mixed/unspecified | ML/Model-based | PPI | Collaborative |
| 2 | Caissie, A. et al. (2025) | Project description / experience report comparing two early-adopter sites implementing ePRO in radiotherapy; narrative with practical lessons. | Canada | To share the patient and community engagement experience of two Canadian institutions that implemented ePRO collection in routine radiotherapy practice. | Mixed/unspecified | Not AI | Co-design/Co-creation | Co-production/Participant-led |
| 3 | Adam R. et al. (2025) | qualitative, co-design project description. | UK | The study aimed to co-design a digital intervention (the SPARC tool) to improve aftercare for people completing potentially curative cancer treatment. The core objectives are: To involve a wide range of stakeholders in fully describing the current problems and gaps in cancer aftercare that might be addressed by digital solutions. To co-design an intervention (the SPARC tool) with these stakeholders. | Colorectal | Unclear/Other | Co-design/Co-creation | Collaborative |
| 4 | Franzoi, M. A. et al. (2025) | Qualitative co-design study using participatory research framework; two phases of focus groups with thematic content analysis; reported per COREQ. | France | Explore preferences, facilitators, and barriers for assessing, communicating, and managing individual risk of long-term toxicities; co-design a pathway to implement risk-prediction algorithms in routine breast-cancer care. | Breast | ML/Model-based | Co-design/Co-creation | Co-production/Participant-led |
| 5 | Kalla M. et al. (2025) | This study is primarily a qualitative research study, It reports the findings of the one-on-one interviews conducted during the project's "generative" phase, which aimed to generate ideas and concepts for the development of a digital health tool. | Australia | In author’s words, The objective of the work was “to generate ideas and concepts, through a co-design paradigm, to inform the  Development of a digital health tool to address the unmet needs of people affected by brain tumors"” | Brain | Unclear/Other | PPI; Co-design/Co-creation | Unclear |
| 6 | Oyewusi, W. et al. (2025) | Qualitative | UK | To describe and report the process and outcomes of a Patient and Public Involvement and Engagement (PPIE) event designed to incorporate patient perspectives into applying NLP to oncology free-text notes, ensuring the research is patient-centred and clinically relevant; specifically, to understand views on three themes, data use, research participation/consent, and research communication, so these insights can shape the project’s design and governance. | Mixed/unspecified | Not AI | PPI | Co-production/Participant-led |
| 7 | Hou SHJ et al. (2025) | Qualitative descriptive study | Canada | To use co-design to identify the priority components to include in a mobile health (mHealth) intervention with young adult survivors of childhood cancer and health care providers. | Leukemia/Lymphoma | Unclear/Other | Co-design/Co-creation | Co-production/Participant-led |
| 8 | Verweij, L. et al. (2023) | Participatory Action Research (PAR) with iterative co-design workshops + questionnaires at ~3 and ≥6 months; descriptive statistics and content analysis. | Netherlands | Assess whether PAR is suitable to improve, implement, and evaluate a real-world eHealth platform (CMyLife) while promoting patient empowerment in CML care. | Leukemia/Lymphoma | Not AI | PAR; Co-design/Co-creation | Co-production/Participant-led |
| 9 | Murphy KM et al. (2025) | Qualitative study using a user-centered design approach. | USA | To engage adolescent and young adult (AYA) cancer survivors and their providers in the concept generation and ideation step of user-centered design to generate ideas for a digital depression self-management tool. | Breast | Unclear/Other | Co-design/Co-creation; UCD/HCD | Collaborative |
| 10 | O’Neill, C. et al.(2024) | Intervention adaptation + formative usability/acceptability optimisation using think-aloud interviews (qualitative, iterative) with end-users; guided by the Person-based Approach. | UK | To adapt a face-to-face, evidence-based, theory-driven educational intervention into an eLearning resource and optimise it for usability and acceptability using a Person-based Approach. | Mixed/unspecified | Not AI | PPI; Person-Based Approach | Collaborative |
| 11 | Leske M, et al. (2022) | Qualitative study. | Australia | To conduct the "ideate and prototype" phases of the co-design process for an online healthy living intervention by presenting wireframes (mock-ups) to stakeholders for feedback. | Breast | Unclear/Other | Co-design/Co-creation | Collaborative |
| 12 | Dang T.H. et al. (2024) | Mixed-methods (design science research methodology and co-design, Unified Theory of Acceptance and Use of Technology (UTAUT) questionnaire) and project description using design science research methodology and co-design. | Australia | Co-design, co-develop, and preliminarily evaluate a Motivational Interviewing Training Platform (MITP)  for oncology Health Care Professionals (HCPs) specifically focused on medication adherence. | Didn’t specify a cancer type | Unclear/Other | Other/Unclear | Co-production/Participant-led |
| 13 | Nuseibeh BZ et al. (2024) | Mixed-methods: Used qualitative thematic analysis from design sessions and quantitative evaluation of the prototype (SUS, Acceptability E-scale). | USA | To engage breast cancer survivors and Acceptance and Commitment Therapy (ACT) content experts in user-centered design (UCD) to develop an mHealth app for stress management. | Breast | Unclear/Other | Co-design/Co-creation; UCD/HCD | Co-production/Participant-led |
| 14 | Fietta, V. et al. (2024) | Co-design + formative usability evaluation (mixed methods): semantic-differential scales, uMARS, and semi-structured interviews across low- and high-fidelity prototype phases. | Italy | Adapt WHO Self-Help Plus (SH+) into a mobile, chatbot-guided intervention and assess usability, attractiveness, and functionality for pregnant women and women with breast cancer, plus clinician/expert stakeholders. | Breast | Not AI | UCD/HCD | Collaborative |
| 15 | Rossi, S. et al. (2024) | qualitative | Not specified | To examine how creating/using digital life stories by people with cancer impacts the research process (ethics, relationships, knowledge production, and dissemination). | Mixed/unspecified | Not AI | Other/Unclear | Co-production/Participant-led |
| 16 | Sien, S-W. (2024) | Co-design + usability study using design thinking (empathize, define, ideate, prototype, test) with RITE rapid iteration; mixed methods (task completion, SUS, interviews with thematic analysis). | Canada | Report user evaluations of a self-/symptom-management app prototype for older adults with cancer and multimorbidity; refine design and assess usability. | Mixed/unspecified | Not AI | Co-design/Co-creation; UCD/HCD | Co-production/Participant-led |
| 17 | Dang, T.H. et al. (2024) | Project description about a mobile solution using Design Science Research methodology (DSRM) that incorporates a mixed methods of evaluation. | Australia | The main objective of this study was to co-design, develop, and preliminarily evaluate an innovative mobile health solution called Safety and Adherence to Medication and Self-Care Advice in Oncology (SAMSON) in order to improve medication adherence  Among people with cancer. | Mixed/unspecified | Unclear/Other | Co-design/Co-creation | Unclear |
| 18 | Young, K. et al. (2024) | Qualitative descriptive UCD/CBPR development with iterative prototyping and think-aloud usability testing; formative evaluation of acceptability/appropriateness/feasibility. | Canada | Culturally adapt the Ned Nurse virtual survivorship app for Chinese Canadian prostate cancer survivors, using UCD + community-based participatory research, while explicitly addressing structural inequities in care. | Prostate | Not AI | UCD/HCD | Collaborative |
| 19 | Kleinlugtenbelt, L. B. et al. (2024) | Mixed-methods, iterative development: Phase 1 online surveys of parents and pediatric physical therapists (PPTs); Phase 2 co-creation sessions with stakeholders to conceptualize a national care network (KinderOncoNet). | Netherlands | Determine the needs of parents and PPTs and the added value of a national care network; use findings to inform the design and development of KinderOncoNet. | Pediatric/AYA | Not AI | Co-design/Co-creation | Co-production/Participant-led |
| 20 | Geerts, P. et al.(2023) | Iterative design-thinking / “action-based” development with stakeholder co-creation, followed by a pilot usability/usage evaluation (SUS + usage logs + qualitative feedback). | Netherlands | Develop a multi-modality e-health application aligned to the MM care pathway; assess usability and end-user experiences. | Didn’t specify a cancer type | Not AI | Co-design/Co-creation | Unclear |
| 21 | Roh, S., & Lee, Y.-S. (2023) | qualitative, | USA | 1) Assess AI women’s knowledge, barriers, and educational needs for breast cancer screening; (2) explore how to design a mobile web app education intervention to improve screening behaviors. | Breast | Not AI | Other/Unclear | Co-production/Participant-led |
| 22 | Santin, O. et al. (2023) | Co-design/development study with qualitative thematic analysis across a six-step co-design and user-testing process. | UK | To rapidly enhance an existing peer-led online resource for cancer carers with a COVID-19 support & information module, using a six-step co-design method. | Mixed/unspecified | Not AI | Co-design/Co-creation | Co-production/Participant-led |
| 23 | Verweij, L. et al. (2023) | Participatory Action Research (PAR) with iterative co-design workshops + questionnaires at ~3 and ≥6 months; descriptive statistics and content analysis. | Netherlands | Assess whether PAR is suitable to improve, implement, and evaluate a real-world eHealth platform (CMyLife) while promoting patient empowerment in CML care. | Leukemia/Lymphoma | Not AI | PAR; Co-design/Co-creation | Co-production/Participant-led |
| 24 | Taramasco C, et al. (2023) | Qualitative case study. | Chile | To identify the information needs of breast cancer patients and health care professionals to integrate into a mobile app that accompanies patients during treatment and allows for reporting adverse symptoms. | Breast | Unclear/Other | Co-design/Co-creation | Consultative |
| 25 | Adler, R. F. et al. (2022) | qualitative | USA | To design an mHealth app for cancer survivors with disabilities that provides interventions to improve quality of life and increase self-efficacy for managing cancer as a chronic condition, using co-design workshops to shape personas, features, and a prototype. | Breast | Not AI | Co-design/Co-creation | Co-production/Participant-led |
| 26 | Wendel, C. et al.(2022) | Mixed-methods descriptive evaluation of stakeholder engagement within a multi-site randomized trial of a telehealth program. Narrative analysis of session notes + 15-item stakeholder survey (descriptive stats). | USA | Describe peer/stakeholder (peer ostomates, ostomy nurses, telehealth engineers) perceptions of engagement and participation in the OSMT telehealth trial. | Mixed/unspecified | Not AI | Other/Unclear | Co-production/Participant-led |
| 27 | Al-Mondhiry, J. et al. (2022) | Participatory design (CPPR) + formative pretest: stakeholder workshops/focus groups → prototype; small patient pretest with semi-structured interviews. QI “Vision” phase. | USA | Use community-partnered participatory research (CPPR) to co-design and pretest a mobile app aligned to palliative-care priorities of clinicians and patients with advanced cancer. | Mixed/unspecified | Not AI | CPPR | Co-production/Participant-led |
| 28 | Dennett, A. M. et al. (2022) | Mixed-methods development/evaluation using experience-based co-design (EBCD) + pilot survey/analytics. | Australia | Develop and evaluate a freely available online Cancer Exercise Toolkit to support exercise professionals working with cancer survivors; assess usability/utility and effect on knowledge, confidence, and behavior. | Mixed/unspecified | Not AI | Co-design/Co-creation | Co-production/Participant-led |
| 29 | Mueller, E. L. et al. (2022) | Mixed methods: co-design + rapid prototyping (phase 1) and alpha testing (phase 2) with semistructured think-aloud interviews and SUS usability survey; NVivo thematic analysis. | USA | Engage caregivers and nurse coordinators (proxies) to co-design and create the Cope 360 app for medical management of a child with cancer; refine via alpha testing; hypothesis that end-user input would lead to necessary refinements before real-world testing. | Pediatric/AYA | Not AI | Co-design/Co-creation; UCD/HCD | Collaborative |
| 30 | Grant, A. R. et al. (2021) | Qualitative co-design (first round) using focus groups and semi-structured interviews with thematic analysis (NVivo); COREQ-guided reporting. | Australia | To understand survivors’, oncology HCPs’, and NGO representatives’ views on (a) what “healthy living” means to survivors and (b) desired content and format of a future online healthy-living program. | Mixed/unspecified | Not AI | Co-design/Co-creation | Collaborative |
| 31 | Singleton, A. et al. (2021) | The study is explicitly identified as a mixed-methods study, which utilized a two-step iterative process to co-design and evaluate a lifestyle-focused text message intervention. | Australia | The main objective of the study is to report the procedures and outcomes of a co-design process used to develop an evidence-based text message program aimed at supporting women’s mental and physical health after breast cancer treatment. | Breast | Unclear/Other | PPI; Co-design/Co-creation | Unclear |
| 32 | Sun, K. et al. (2021) | Project description / development study using a multidisciplinary co-design approach with mixed methods (systematic review, interviews, focus groups, eHNA data, workshops), plus a pilot of the MVP. | UK | To describe the multidisciplinary, theory-based co-design process and resulting features of a digital health intervention (DHI) intended to improve experience of care and reduce unmet needs among oesophageal cancer patients. | Esophageal | Not AI | PPI; Co-design/Co-creation; UCD/HCD | Co-production/Participant-led |
| 33 | Lai‑Kwon J, et al. (2024) | Qualitative Co-design Study. co-design workshops. Inductive Thematic Analysis | Australia | To create a new digital health system prototype and define the associated workflow, rather than testing a hypothesis or measuring outcomes on a large scale. aimed to co-design an Electronic Patient-Reported Outcome (ePRO) symptom monitoring system prototype integrated with the Electronic Medical Record (EMR) for Immune Checkpoint Inhibitor (ICI) toxicities | Prostate | ML/Model-based | Co-design/Co-creation; UCD/HCD | Collaborative |
| 34 | Pozzar, R. A. et al. (2025) | Mixed-methods development/usability study using design thinking + cognitive interviews and surveys (SUS, AIM, IAM) to assess a portal/EHR-integrated agenda-setting tool. | USA | Assess usability and acceptability of CASI and identify barriers/facilitators to implementation to inform a future pilot RCT. | Gynecologic | Not AI | Other/Unclear | Collaborative |
| 35 | Grindell, C. et al. (2020) | Service improvement / development study using creative co-design/co-production to generate a prototype patient decision support tool. | UK | To co-produce an initial prototype decision support tool (“My Pleural Effusion Journey”) to support shared treatment decisions for malignant pleural effusion (MPE). | Breast | Not AI | Co-design/Co-creation; UCD/HCD | Co-production/Participant-led |
| 36 | Bak, M. et al. (2025) |  | UK | 1) Introduce an embedded-review approach to surface ethics within a large data-driven oncology consortium; (2) identify key ethical challenges for designing and developing DSTs and redesigning care paths; (3) sensitize teams and create a shared ethical framework/vocabulary for action. | Breast | ML/Model-based | PPI; UCD/HCD | Co-production/Participant-led |
| 37 | Jibb, L. A. et al. (2024) | Co-design + iterative usability testing (mixed methods): think-aloud sessions, field notes, System Usability Scale (SUS), and semi-structured interviews with thematic analysis, across 3 cycles until saturation. | USA | Use iterative rounds of mixed-methods user testing and software modification to refine PainCaRe, a real-time pediatric cancer pain management app for parents of children aged 2–11, to be easy to use/understand, efficient, acceptable, and clinically useful. | Pediatric/AYA | Not AI | Co-design/Co-creation | Collaborative |
| 38 | Xiong, S. et al. (2025) | Mixed-methods D&I pilot (qualitative interviews + quantitative web analytics/survey). | USA | Determine the most effective dissemination and implementation strategies for a culturally tailored HPV eHealth website in schools and primary care clinics using community-engaged methods (CBPR). | Pediatric/AYA | Not AI | Other/Unclear | Co-production/Participant-led |
| 39 | Wang, Y. et al. (2025) | Mixed method: quantitative structured expert elicitation (producing pooled beta distributions for three parameters) and qualitative semi-structured interviews with content analysis | Australia | To conduct and evaluate the acceptability of a co-designed structured expert elicitation (SEE) with clinicians, eliciting expert opinions on three uncertain model parameters for a decision-analytic model in exercise oncology (CVD risk reduction from exercise, clinician referral probability, and patient uptake), and to collect qualitative feedback to refine future SEE practice. | Mixed/unspecified | Not AI | Co-design/Co-creation | Collaborative |
| 40 | Perry, L. M. et al. (2022) | Protocol: stakeholder co-design + longitudinal demonstration trial (pre–post) embedded in EHR; implementation outcomes planned. | USA | (1) Co-design a PRO dashboard that displays symptoms/HRQoL with clinical data in the EHR; (2) conduct a longitudinal trial to see if the dashboard improves shared decision-making and disease-management outcomes. | Lung | Not AI | Co-design/Co-creation | Co-production/Participant-led |
